# Supplementary material for: Use of a new micropattern tape method to detect chirality shifts in differentiating C2C12 cells
Source: PLoS One. 2025 Dec 4;20(12):e0338032. doi: 10.1371/journal.pone.0338032 (PMC12677580; doi:10.1371/journal.pone.0338032)
Supplement: S1 Appendix — (DOCX) [file pone.0338032.s001.docx]

**S1 Appendix.** **Optimization of cell seeding density for the tape method.**


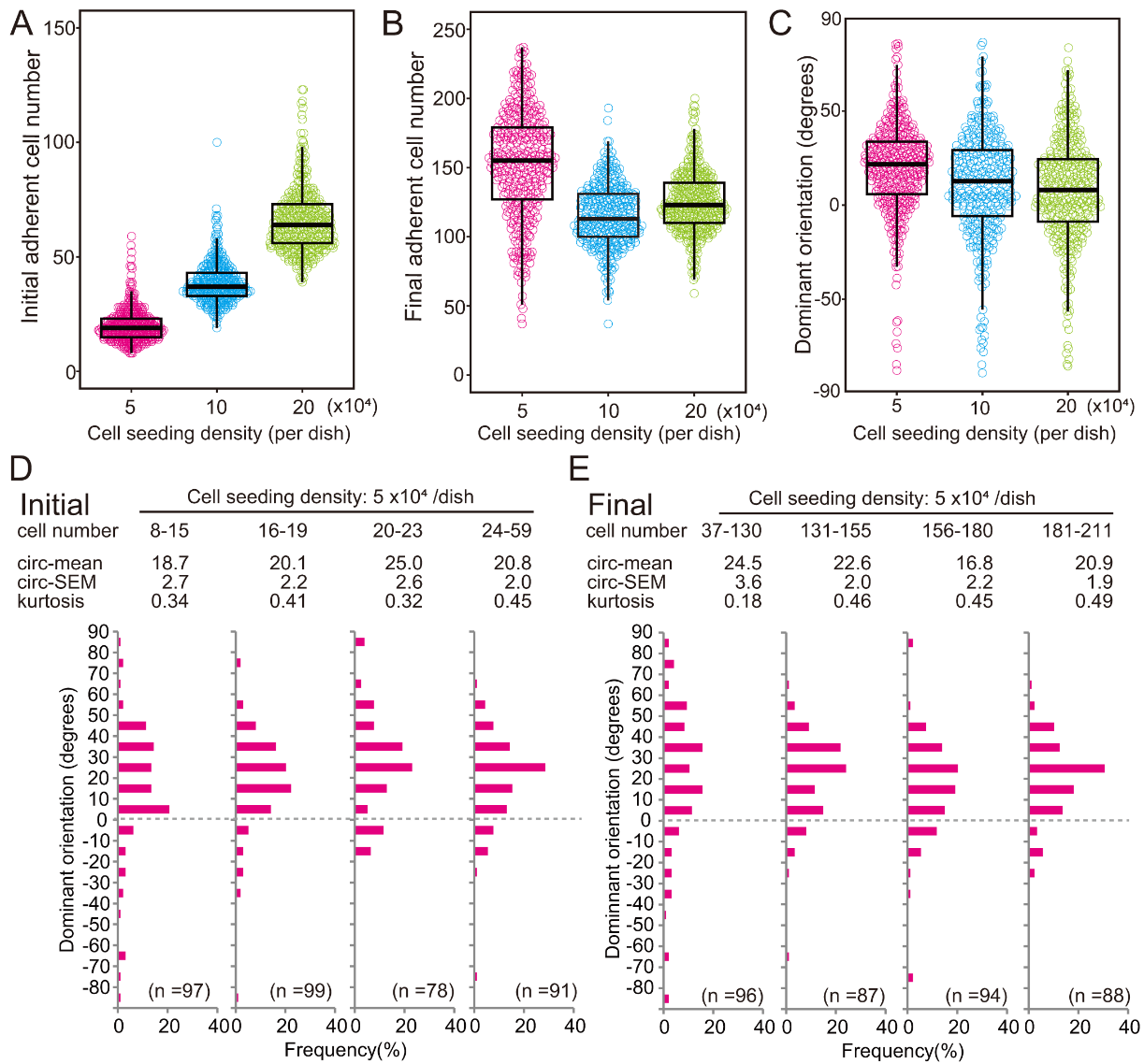
 To determine the optimal seeding density of MC3T3-E1 cells to facilitate analysis of cell alignment on micropatterns, we asked whether varying cell seeding density might alter distribution of dominant orientations of cells. Suspensions of trypsinized cells were first stained 30 minutes with Hoechst 33342 (Nacalai, 04929-82, 5 μg/mL) before plating onto the micropattern at increasing densities of 5x10^4^, 1x10^5^, and 2x10^5^ cells per dish.  Once cells adhered, the medium was refreshed. Then, immediately afterwards, we determined the initial number of adherent cells in each micropattern by live cell imaging using Thunder Imaging Systems (Leica) and by counting nuclei (Fiji). As expected, a greater number of cells adhered to the micropattern as cell seeding density increased (Fig S1A). Cells seeded at different densities were then cultured for different durations until they reached confluency, when we calculated the final number of adherent cells in each micropattern by counting nuclei (Fig S1B). To determine potential effects of cell seeding density on cell alignment, we evaluated the dominant cell orientation on each micropattern among the 3 cell densities and found that the bias in distribution of the dominant orientation was greater at lower initial density (Fig S1C), suggesting that chirality is more readily observed when cells are less crowded and that higher densities may obscure visibility of the dominant orientation. Dominant orientation data shown in Fig S1C (red, 5x10⁴ cell seeding density per dish) were further divided into four groups according to the initial (Fig S1D) or final (Fig S1E) number of cells in the micropatterns.

Fig. S1. Effect of cell seeding density on a chirality assay. (A) Initial number of adherent cells, (B) final number of adherent cells, and (C) analysis of dominant orientation. Numbers of micropatterns plotted were 365, 325, and 359, and culture duration was 6, 4, and 3 days, for cell seeding densities 5 x 10^4^ (red), 1 x 10^5^ (blue), and 2 x 10^5^ (green) per dish, respectively. Boxplot indicates the interquartile range (IQR) with median (horizontal line), and whiskers up to 1.5× IQR. (D) Orientation histograms of the four groups according to the initial cell number on each micropattern after seeding 5x10⁴ cells (Fig S1C, red). (E) Orientation histograms of the four groups according to the final cell number of on each micropattern after seeding 5x10⁴ cells (Fig S1C, red).
