# Supplementary material for: Use of a new micropattern tape method to detect chirality shifts in differentiating C2C12 cells
Source: PLoS One. 2025 Dec 4;20(12):e0338032. doi: 10.1371/journal.pone.0338032 (PMC12677580; doi:10.1371/journal.pone.0338032)
Supplement: S2 Appendix — (PDF) [file pone.0338032.s002.pdf]

S2 Appendix.

Table S2. List of primers used in this study

Gene	Primer name	5'-Sequence-3'	amplicon
Rpl13a	mRpl13a_S	CACCCACCTGACCAAGTACCC	118
Rpl13a	mRpl13a_AS	TGCTTTGTGGTCTGCTGGGAAG	
Actn1	mActn1_S	TGACATAGGAAAATAACCAAGCAC	140
Actn1	mActn1_AS	CTGATGAGAGTACCCACCTACC	
Actn2	mActn2_S	CTAGAGAGACCGCAGACACCG	79
Actn2	mActn2_AS	CGACGAAGCTCCTCTGCCAAG	
Pfn1	mPfn1_S	CCCCATTTCCCTTATTGCTGCC	90
Pfn1	mPfn1_AS	CACAGGAGGGGGTATGGGTAG	
Fscn1	mFscn1_S	AGAAAGCCGGATGTGCCCCTC	119
Fscn1	mFscn1_AS	AGGAGCGAGACACCCAGAGTG	
Fmn1	mFmn1_S	TTGCAGAAGTCTTAAGTCCACCC	136
Fmn1	mFmn1_AS	GGGATCACCATGTACCTCTTTTC	
Runx2	mRunx2_S	CGAATGGCAGCACGCTATTAAATC	127
Runx2	mRunx2_AS	CCGCCAAACAGACTCATCCATTC	
Alpl	mAlpl_S	CCATAGTCACGGCCAGTCCTC	102
Alpl	mAlpl_AS	GTAGACAGCCAACCCCGGAGG	
Myog	mMyog_S	CCTTGCTCAGCTCCCTCAACC	101
Myog	mMyog_AS	GGCGCTGTGGGAGTTGCATTC	
Ckm	mCkm_S	ACAGCACAGACAGACACTCAGG	136
Ckm	mCkm_AS	GTTGTGCTTGCTGAGGTCTGGG	
Myh1	mMyh1_S	AACTGCAATCAAAGGTCAAGGCC	93
Myh1	mMyh1_AS	GCTGGATCTTGCGGAATTTGGC	
