# Supplementary material for: Use of a new micropattern tape method to detect chirality shifts in differentiating C2C12 cells
Source: PLoS One. 2025 Dec 4;20(12):e0338032. doi: 10.1371/journal.pone.0338032 (PMC12677580; doi:10.1371/journal.pone.0338032)
Supplement: S3 Appendix — (DOCX) [file pone.0338032.s003.docx]

**
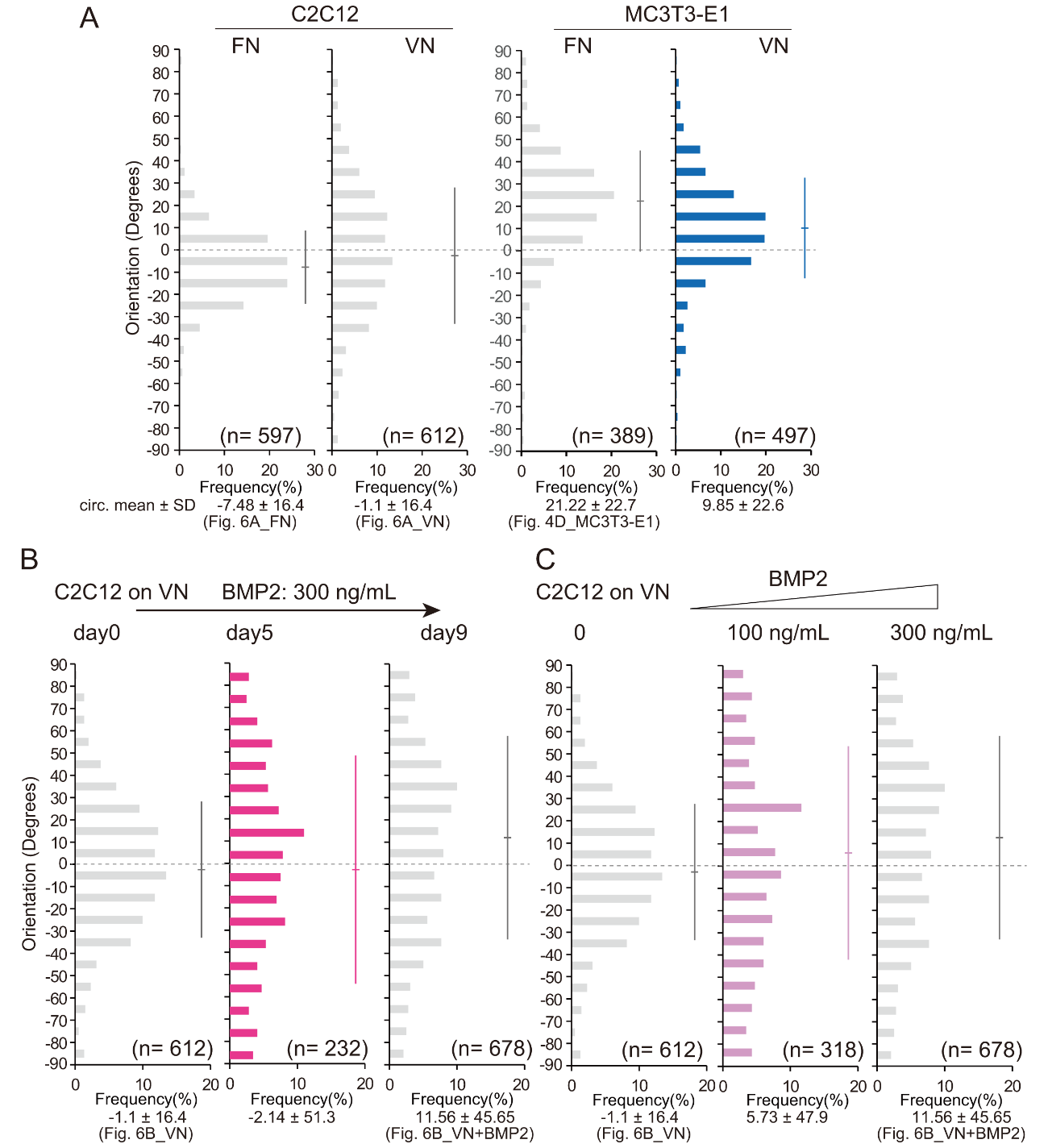
S3 Appendix.**

**S3 Appendix.** **Effect of coating substrates and conditions of BMP2 treatment on the dominant orientation.** (A) Dominant orientation of C2C12 and MC3T3-E1 cells on micropattern coated with fibronectin (FN) or vitronectin (VN). (B) Dominant orientation of C2C12 cells at different times after BMP2 stimulation. (C) Dominant orientation of C2C12 cells at day 9 after stimulation with different BMP2 concentration. Vertical bars indicate circular mean ± SD.
