# Supplementary material for: Use of a new micropattern tape method to detect chirality shifts in differentiating C2C12 cells
Source: PLoS One. 2025 Dec 4;20(12):e0338032. doi: 10.1371/journal.pone.0338032 (PMC12677580; doi:10.1371/journal.pone.0338032)
Supplement: Attachment — Submitted filename: Manuscript_ PONE-D-25-25096.docx [file pone.0338032.s005.docx]

**Manuscript Review**

**Manuscript: PONE-D-25-25096**

**Title: Use of a New Micropattern Tape Method to Detect Chirality shifts in differentiating C2C12 Cells**

**General Comments**: Chirality is a property of cells demonstrating right and left behavior in terms of morphology and organization. The present paper develops a new method to pattern small regions of cells applied in rectangular micro patterns for chirality analysis. The authors develop a unique method utilizing a tape system which enables application of a range of extracellular matrix proteins while creating gap regions between the applied cell areas. They also have developed a sophisticated optical microscopic analysis system to determine orientation and a processing method for quantitation of images obtained. Using this method they look at the impact of an exogenous agent - bone morphogenic protein on alteration of chirality of two cell lines C2C 12 and MC3T3E1. They specifically show that the system can detect plasticity of cell chirality *in vitro*. The paper in general is well written is clear and describes the unique method. There are few areas which may be addressed to strengthen the paper

**General issues to be discussed:**

1. Provide a bit more in the discussion of other existing *in vitro* fabrication methods that other investigators are using and provide a bit more point by point detail of why your method is better.

2. Please add detail as to the nature of the tape, its constituents, its composition and the nature of the adhesive.

3. Please add detail as to the uncoated film bottom dish. What is this material? Is this available for other investigators. In general, both of these details in points #2 and #3 above are important if a method is going to be more universally applied by others.

4. For figure 5 where there is reference to a range of extracellular matrix proteins please clarify if this involved the same general coating technique of the dish/microislands or is this different?? This is confusing to the reader.

5. In the discussion at the beginning in the first sentence it states, “cell adhesive fibronectin patterns”. Related to point #4 if this is only for fibronectin that is fine. However, if other adhesive proteins are being utilized here as well this is confusing to the reader and needs to be clarified.

6. On page 18 lines 390 through 392 need to be clarified. Specifically, the issue of what is being adhered to what, and to prevent adhesion to uncoated areas is unclear to the reader - please explain.

7. In the manuscript the word “holes” was used to designate the gap areas. Would use a different term such as uncoated area or region. For most readers holes will convey a mental image of something round rather than the rectangular regions you actually create.

8. would provide a bit more in the discussion re the sensitivity of this techniques and the optical/image analysis system you developed. How sensitive is this? How accurate? How does this compare to other systems.

9. As to the biological findings of chirality changing with differentiation and cell aging or ECM effects – would expand on this a bit with discussions as to what is known in the literature and how do your findings conform or extend the body of knowledge.
